# Identification of a robust signature for clinical outcomes and immunotherapy response in gastric cancer: based on N6-methyladenosine related long noncoding RNAs

**DOI:** 10.1186/s12935-021-02146-w

**Published:** 2021-08-16

**Authors:** Tenghui Han, Dong Xu, Jun Zhu, Jipeng Li, Lei Liu, Yanchun Deng

**Affiliations:** 1 Xijing Hospital, Airforce Medical University, Xi’an, 710032 China; 2 School of Clinical Medicine, Xi’an Medical University, Xi’an, 710032 China; 3 Daping Hospital, Army Medical University, Chongqing, 400042 China

**Keywords:** m^6^A modification, Gastric cancer, Immune checkpoint blockers, LncRNA, FTO

## Abstract

**Background:**

Gastric cancer (GC) is a globally prevalent cancer, ranking fifth for incidence and fourth for mortality worldwide. The N6-methyladenosine (m^6^A) related long noncoding RNAs (lncRNAs) were widely investigated in recent studies. Nevertheless, the underlying prognostic implication and tumor immune mechanism of m^6^A-related lncRNA in GC remain unknown.

**Methods:**

We systematically assessed the m^6^A modification expression of 407 GC clinical samples based on 23 m^6^A regulators and comprehensively associated these genes with lncRNAs. Then, we constructed a m^6^A-related lncRNA prognostic signature (m^6^A-LPS) to evaluate both status and prognosis of the disease. Immune-related mechanisms were explored via dissecting tumor-infiltrating cells as well as applying tumor immune dysfunction and the exclusion algorithm. Furthermore, we validated the latent regulative mechanism of m^6^A-related lncRNA in GC cell lines.

**Results:**

The m^6^A-LPS containing nine hub lncRNAs was built, which possessed a superior capability to predict the outcomes of GC patients. Meanwhile, we found an intimate correlation between the m^6^A-LPS and tumor infiltrating cells, and that the low-risk group had a higher expression of immune checkpoints and responsed more to immunotherapy than the high-risk group. Clinically, these crucial lncRNAs expression levels were verified in ten pairs of GC samples. In in vitro experiments, the abilities of migration and proliferation were significantly enhanced via downregulating the lncRNA AC026691.1. Both migrative and proliferative capabilities of tumor cells were significantly enhanced via downregulating the lncRNA AC026691.1. in vitro.

**Conclusions:**

Collectively, the m^6^A-LPS could provide a novel prediction insight into the prognosis of GC patients and serve as an independent clinical factor for GC. These m^6^A-related lncRNAs might remodel the tumor microenvironment and affect the anti-cancer ability of immune checkpoint blockers. Importantly, lncRNA AC026691.1 could inhibit both migration and proliferation of GC by means of FTO regulation.

**Supplementary Information:**

The online version contains supplementary material available at 10.1186/s12935-021-02146-w.

## Introduction

Gastric cancer (GC) is recognized as the fifth most common malignant tumor worldwide, and over one million new cases are diagnosed annually. In light that GC is frequently diagnosed at an advanced age, 769,000 patients died globally in 2020, ranking fourth in mortality worldwide [1]. Despite the fact that advanced GC patients could be treated with chemotherapy clinically, the curative effect is poor with median survival being less than 1 year [2]. In contrast to chemotherapy, immunotherapy has been authenticated to have durable curative effect and marked clinical benefit amidst a limited percentage of GC patients [3–5].

In the living organism, there exist more than 100 RNA epigenetics modifications, amidst which N6-methyladenosin (m^6^A) is the most prevalent and abundant form of post-transcriptional modification for mRNA, miRNA as well as long noncoding RNA (lncRNA) [6, 7]. The process of m^6^A methylation is intimately associated with three categories of molecular compositions: “writers” (m^6^A methyltransferases), “readers” (m^6^A recognition factors) and “easers” (m^6^A demethylase) [8]. Recently, convincing evidence has identified that there was an intimate relationship between m^6^A modified lncRNAs and neoplastic progression [9, 10].

Recently, immunotherapy has been gradually identified as an indispensable method for cancer treatment and demonstrated an irreversible trend [11]. Immune checkpoint blockers (ICB) therapy is defined as a kind of specialized anti-tumor immunotherapy targeting immune checkpoint proteins, including PD-1 and CTLA-4 [12]. Immune checkpoint proteins are highly relevant to initiation of immunocyte signaling pathways, which could be manipulated by tumor cells to escape immune response and form tumor microenvironment (TME) that is beneficial to neoplastic development [13–15]. Furthermore, multitudes of investigations have indicated that lncRNAs play non-negligible roles in cancer immunity [16, 17]. Nonetheless, the underlying prognostic value and tumor immune mechanism of m^6^A-related lncRNA in GC remain unclear. Thus, it is of paramount significance to search for biomarkers that could serve as potential treatment targets and explore tumor immunotherapy from the mechanistic perspective of m^6^A modification.

**Fig. 1:**
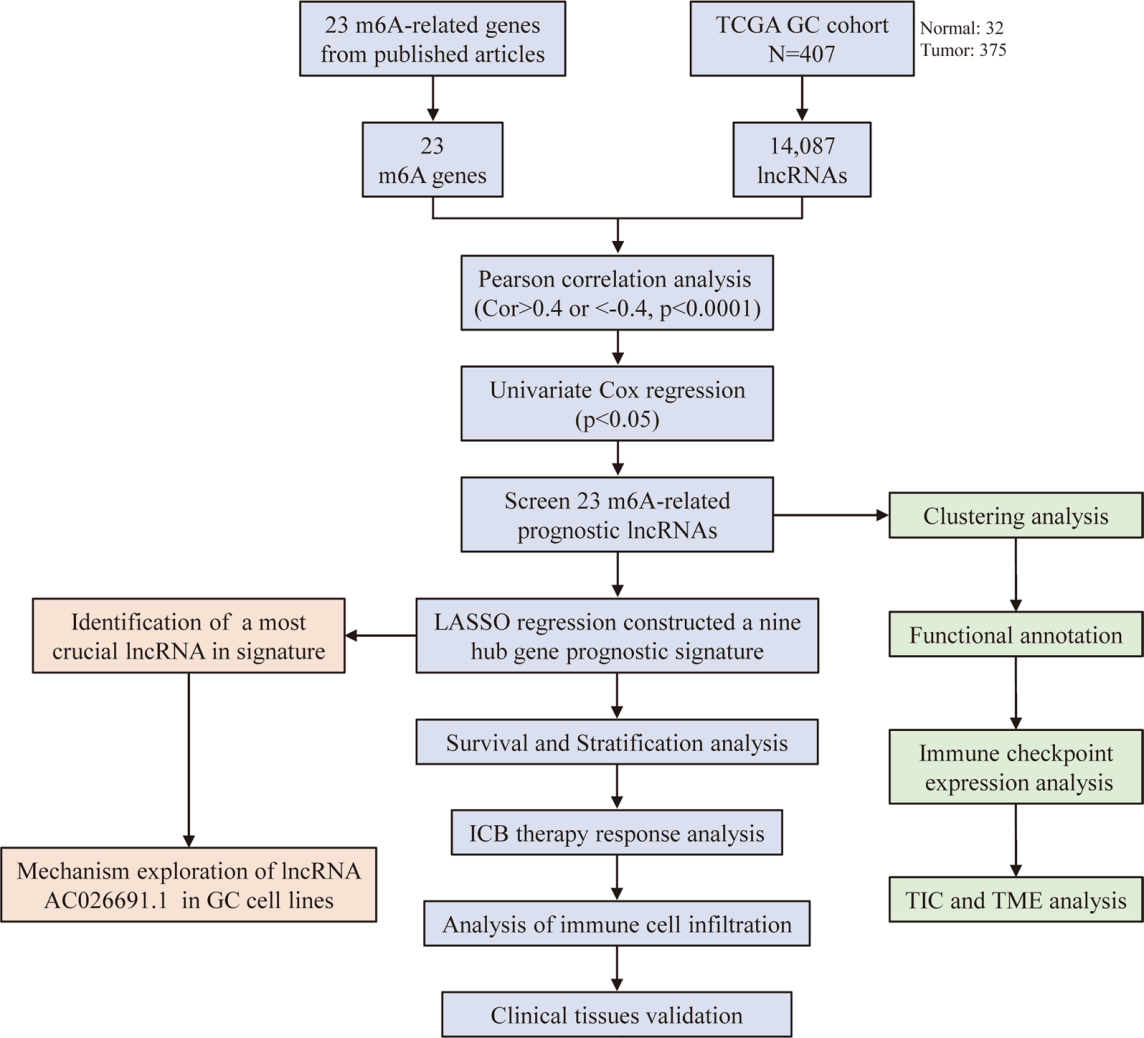
The workflow of our study

Our present study successfully identified m^6^A-related lncRNAs and for the first time built a novel prognosis-related lncRNA signature which is superior in predicting the survival of GC patients. Next, by exploring the direct crosstalk between m^6^A-related lncRNAs and TME, we found that m^6^A-related lncRNAs could potentially influence cancer immunotherapy via remodeling of TME and alteration of ICB sensitivity. More importantly, by doing plentiful in vitro experiments, we demonstrated that lncRNA AC026691.1 could function as a tumor suppressor gene in GC, which has an intimate association with m^6^A eraser, namely fat mass and obesity-associated protein (FTO) gene. The workflow of our study was shown in Fig. 1.

## Materials and methods

### GC dataset acquisition

Transcriptome RNA sequencing data of 32 paracancerous and 375 cancerous GC samples were acquired from The Cancer Genome Atlas (TCGA) database (https://portal.gdc.cancer.gov/). Clinical data (age, sex, tumor differentiation grade and TNM stage) of patients were directly retrieved from TCGA.

### m^6^A-related prognostic lncRNAs

According to previous reports [8, 18], 23 m^6^A-related genes were obtained involving eight writers, 13 readers, and two erasers. For further analysis, we identified m^6^A-related lncRNAs by employing Spearman's test with an absolute value of > 0.4 or < -0.4, P < 0.0001. To screen m^6^A-related prognostic lncRNAs, we then conducted the univariate Cox regression analysis with the statistically significant criteria being P < 0.05.

### Clustering analysis

Based on the expression of m^6^A-related prognostic lncRNAs, we performed unsupervised clustering analysis of GC samples from TCGA database. Additionally, to explore the survival difference, we conducted survival analysis, plotted Kaplan–Meier (K-M) curves and validated both with the log-rank test. Furthermore, we carried out the correlation analysis of clinical characteristics and got the clustering results.

### Functional annotation

Gene set enrichment analysis (GSEA) was utilized to explore potential functional pathways. By employing the GSEA software, we analyzed two extensively applied gene sets [h.all.v7.2.symbols.gmt (cancer hallmarks) and c7.all.v7.2.symbols.gmt (Immunologic signatures)], which were downloaded from the Molecular Signatures Database. To obtain a standardized enrichment score for each analysis, we performed gene set permutations a thousand times. A nominal P < 0.05 was deemed as statistically significant.

### Tumor-infiltrating immune cell profiling and TME

We evaluated the tumor-infiltrating immune cell (TIC) abundance profile of GC samples and immune-related biological functions by the CIBERSORT algorithm in the “gsva” R package. The 24 categories of TICs include 18 T-cell subtypes and six other kinds of immune cells. The stromal, immune, and ESTIMATE scores of each sample, which reflect the ratio of the immune/stromal components in TME, were also acquired.

### Establishment and demonstration of m^6^A-LPS

The least absolute shrinkage and selection operator (Lasso) regression was conducted to establish m^6^A-LPS via utilizing the “glmnet” R package. Responding coefficients (β) of m^6^A-LPS were verified. Besides, m^6^A-LPS was calculated by the following equation: Risk scores = $\sum (\mathrm{exp}\left(lncRNAs\right)*\beta )$, where exp indicated RNA expression in GC samples, and *β* represented its coefficients. Additionally, scatter diagrams were performed based on the risk score of each sample.

The areas under the curve (AUC) of the receiver operating characteristic (ROC) curve were applied to estimate the predictive value of the m^6^A-LPS. Besides, we conducted both univariate and multivariate cox regression analysis. Stratification analysis was utilized to evaluate the predictive survival value of m^6^A-LPS in disparate clinicopathological sections. Furthermore, we employed SRAMP (a computational predictor of mammalian m6A site) (http://www.cuilab.cn/sramp/) to explore the potential m^6^A modification positions for these corresponding lncRNAs [19].

### Prediction of immunotherapeutic response

Expression of immune checkpoints is intimately correlated with immunization treatment response. Eight critical immune checkpoints comprising programmed death 1 (PD‐1) [14] and its ligand 1 (PD‐L1) [15] and ligand 2 (PD‐L2) [20], indoleamine 2,3-dioxygenase 1 (IDO1) [21], cytotoxic T‐lymphocyte antigen 4 (CTLA‐4) [22], T‐cell immunoglobulin domain, mucin domain‐containing molecule‐3 (TIM‐3) [23], lymphocyte-activation gene 3 (LAG3) and T cell immunoreceptor with Ig and ITIM domains (TIGIT) pathways [24] were investigated to analyze the correlation of immune checkpoints with m^6^A-LPS. Herein, both tumor immune dysfunction and exclusion algorithm and subclass mapping were utilized to predict clinical treatment responses to ICBs [25].

### Collection of clinical samples

Ten pairs of cancerous and paracancerous tissue samples were collected from GC patients who have received surgeries in Xijing Digestive Hospital. All procedures incorporating human participants were in accordance with the Declaration of Helsinki (as revised in 2013). Besides, our present study was approved by the Committee for Ethics in Xijing Hospital. Informed consent was obtained from each patient.

### Cell culture and transfection

Human GC cell lines SGC-7901 and BGC-803 were acquired and then cultivated in Roswell Park Memorial Institute1640 medium (Gibco, USA) supplemented with 10% fetal bovine serum (FBS, Gibco, USA). Small interference RNAs (siRNA) were designed and generated by Sangon Biotech (Shanghai, China). Cell transfection was mediated by lipofectamine 3000 (Invitrogen, USA). Interference sequences were listed in Additional file 1: Table S1.

### Cell migration and viability assay

The wound-healing assay was utilized to assess the migration capability of GC cells. The transfected cells were cultured in 6-well plates (5 × 10^5^ cells per well). Using 200 µL pipette tips, we generated a linear wound across the cell monolayer for each well. Then, after incubation in a serum-free medium for 24 and 48 h respectively, wound monolayer images were captured under the inverted microscope.

Via performing the Cell Counting Kit-8 (CCK-8) assay, we examined the proliferation rates of GC cells. GC cell lines were transfected with siRNA for 36 h. Afterwards, cells (3 × 10^3^ cells per well) were cultivated in 96-well plates for 24 h. Before absorbance measurement at 450 nm in Bio-RAD (Hercules, USA) Microplate Reader, each well was incubated with 10 μL CCK-8 solution while growth graphs were formatted with GraphPad Prism 5.1.

### Dot blot assay

With assistance of TRIzol reagents, total RNA samples were extracted from SGC-7901 and BGC-803 cells respectively. Utilizing Hieff NGS® mRNA Isolation Master Kit (Yeasen Biotechnology; Shanghai, China), the mRNA was further separated and purified. The isolated mRNA was denatured under vacuum conditions at 65 ℃ for 5 min. Afterwards, the nylon membrane (Amershan; RPN303B; USA) was prepared in saline sodium citrate buffer for 20 min and fixed on the m^6^A beater (Bio-Rad; Shanghai, China) for sample addition. After taking advantage of the ultraviolet ray to cross-link, the membrane was stained by methylene blue solution to examine RNA loading. The membrane was sealed with 5% skimmed milk for 1 h, incubated with the antibody against m^6^A (Abcam; ab151230; 1:1000) at 4℃ for overnight, and further incubated with secondary antibody at room temperature for 1 h. Ultimately, the dots were detected by employing Tanon 5500 chemiluminescence imaging system (Tanon Science & Technology; Shanghai, China).

### Western blot analysis

GC cells were directly lyzed in RIPA Lysis Buffer (Sigma, USA). Moreover, proteins were separated by utilizing SDS polyacrylamide gels, transferred by employing polyvinylidene fluoride membranes and sealed with 5% nonfat milk. The membranes were incubated with primary antibodies against FTO (Proteintech; 27226-1-AP; 1:1000) and β-actin (Boster; BM0627; 1:1000) at 4 °C for overnight. Afterwards, second antibodies were incubated for 1 h at room temperature. Ultimately, we took advantage of the ECL chemiluminescent regents and visualized by utilizing Tanon 5500 to quantify proteins.

### Quantitative real-time polymerase chain reaction

To detect the expression of m^6^A-related lncRNAs, total RNA was extracted from clinical GC samples by TRIzol reagent. According to the Reverse Transcription Kit manufacturer’s protocol, total RNA was reverse transcribed into cDNA by utilizing PrimeScript RT Master Mix (TaKaRa, Tokyo, Japan). Then, we employed quantitative real-time polymerase chain reaction (qRT-PCR). Primer sequences for qRT-PCR in our study were listed in Additional file 2: Table S2.

### Statistical analysis

All statistical analyses were conducted in R software (version 3.63). P-value < 0.05 was considered to be statistically significant. K-M curve analysis with a log-rank test was utilized to compare overall survival (OS) between diverse subgroups. Mann–Whitney test with adjusted P values was employed to compare either ssGSEA scores of immune cells or functions of the two groups. Both univariate and multivariate Cox analysis was utilized to identify independent clinical prognostic factors.

## Results

### m^6^A-related lncRNAs in GC

**Fig. 2:**
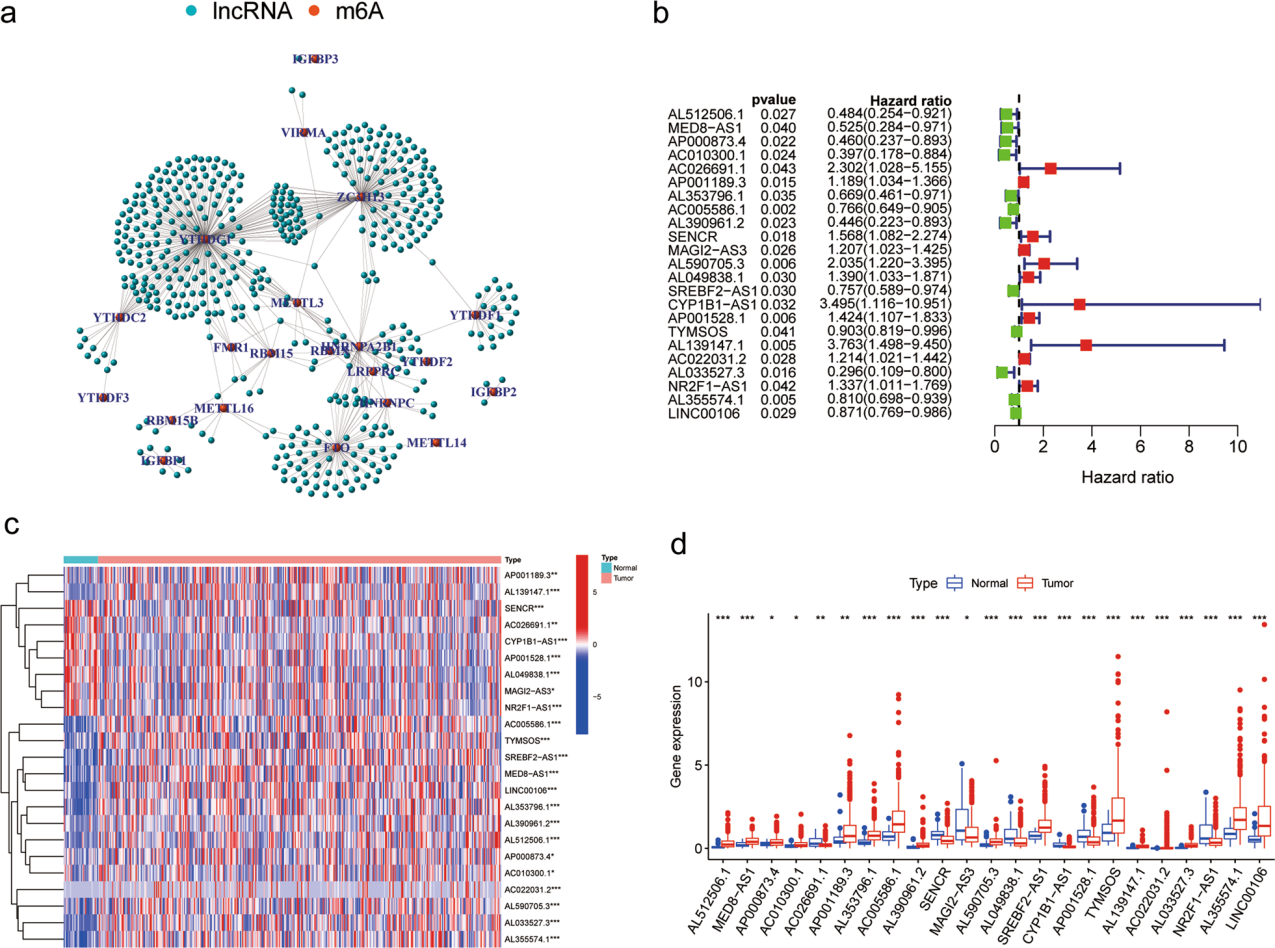
Prognostic value of m^6^A-related lncRNAs in GC. **a** Co-expression network diagram of the 23 m^6^A-related genes (red) and 491 lncRNAs (green). **b** Forest plot of the univariable regression analysis results of the 23 selected m^6^A-related lncRNAs. **c** Heatmap of 23 m^6^A-related lncRNA expression levels in 32 normal and 375 tumor GC samples from the TCGA. **d** Differentially expression analysis of 23 m^6^A-related lncRNAs in normal and tumor samples. P < 0.05 *; P < 0.01**; P < 0.001***. m^6^A, N6-methyladenosin; lncRNA, long noncoding RNA; GC, Gastric cancer

We defined m^6^A-related lncRNAs as those that were significantly correlated (P < 0.0001, |Cor|> 0.4) with m^6^A-related genes. Ultimately, 491 m^6^A-related lncRNAs were obtained. Furthermore, co-expression network of the m^6^A-related genes and lncRNAs was plotted (Fig. 2a). According to univariate Cox regression analysis (Fig. 2b), we obtained 23 m^6^A-related prognostic lncRNAs (P < 0.05) (Additional file 3: Table S3). Based on 32 normal samples and 375 GC samples from TCGA dataset, differential expression analysis of these m^6^A-related lncRNAs was made. Amidst 23 m^6^A-related lncRNAs of tumor samples, 15 lncRNAs exhibited significantly higher expression degrees (P < 0.05) while 8 lncRNAs demonstrated relatively lower expression levels (P < 0.05) (Fig. 2c, d) when compared with normal samples.

### Cluster analysis of m^6^A-related prognostic lncRNAs

**Fig. 3:**
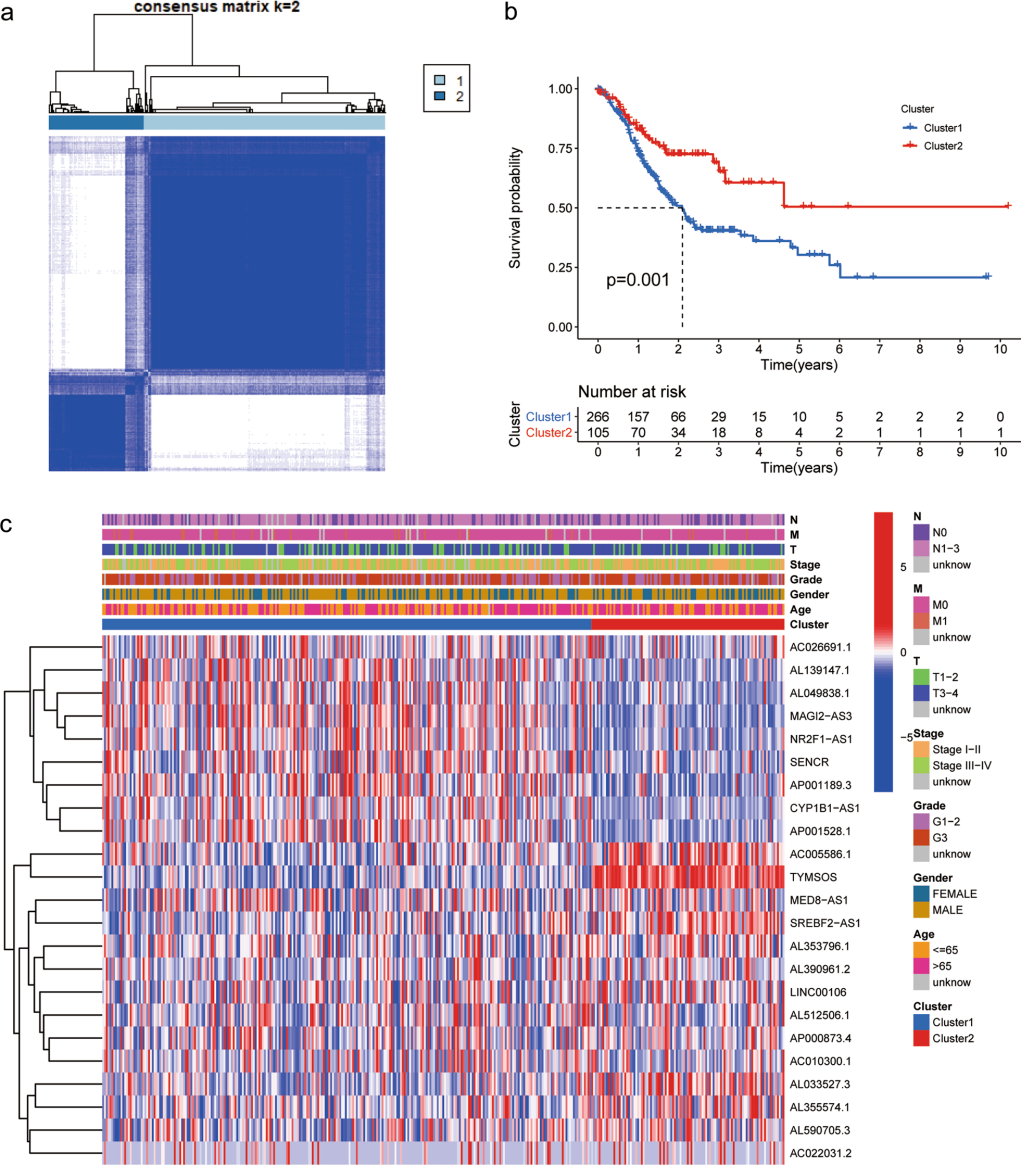
Unsupervised clustering of m^6^A-related prognostic genes. **a** Consensus clustering matrix for k = 2. **b** Survival analysis between two clusters. **c** The correlation of two clusters and clinical characteristics. T stands for T classification, N stands for N classification, and M stands for M classification in TNM staging system. m^6^A, N6-methyladenosin

According to the expression profiles of m^6^A-related prognostic lncRNAs, we conducted unsupervised clustering to analyze the GC samples from TCGA dataset and divided samples into different subtypes. As exhibited in Fig. 3a, k = 2 was the most optimized selection. In order to further explore the relation between clustering result and clinical prognosis, we made survival analysis to compare the OS of GC patients between two subtypes. The consequence demonstrated that OS rate of cluster1 was inferior to that of cluster2 (P = 0.001) (Fig. 3b). Moreover, correlations between the cluster analysis and other clinical parameters, including age, gender, TNM stage, tumor stage and tumor grade, were plotted in the heatmap (Fig. 3c).

### Functional enrichment analysis

In consideration of the favorable clustering result of OS for GC patients, we conducted GSEA between cluster1 and cluster2 to explore the potential biofunction of m^6^A-related lncRNAs. GSEA results indicated that several different tumor hallmarks were significantly enriched in two clusters, such as cell cycle, P53 signaling pathway, ECM receptor interaction and MAPK signaling pathway (P < 0.05) (Additional file 4: Figure S1a–d). Meanwhile, we found that certain immunity pathways were intimately associated with mast cells, Dendritic cells (DC), Natural Killer (NK) cells and T cells (P < 0.05) (Additional file 4: Figure S1e–h). Consequently, these results revealed that m^6^A-related prognostic lncRNAs were highly relevant to tumorigenesis and immune pathways.

### Immune analysis of m^6^A related prognostic lncRNAs

**Fig. 4:**
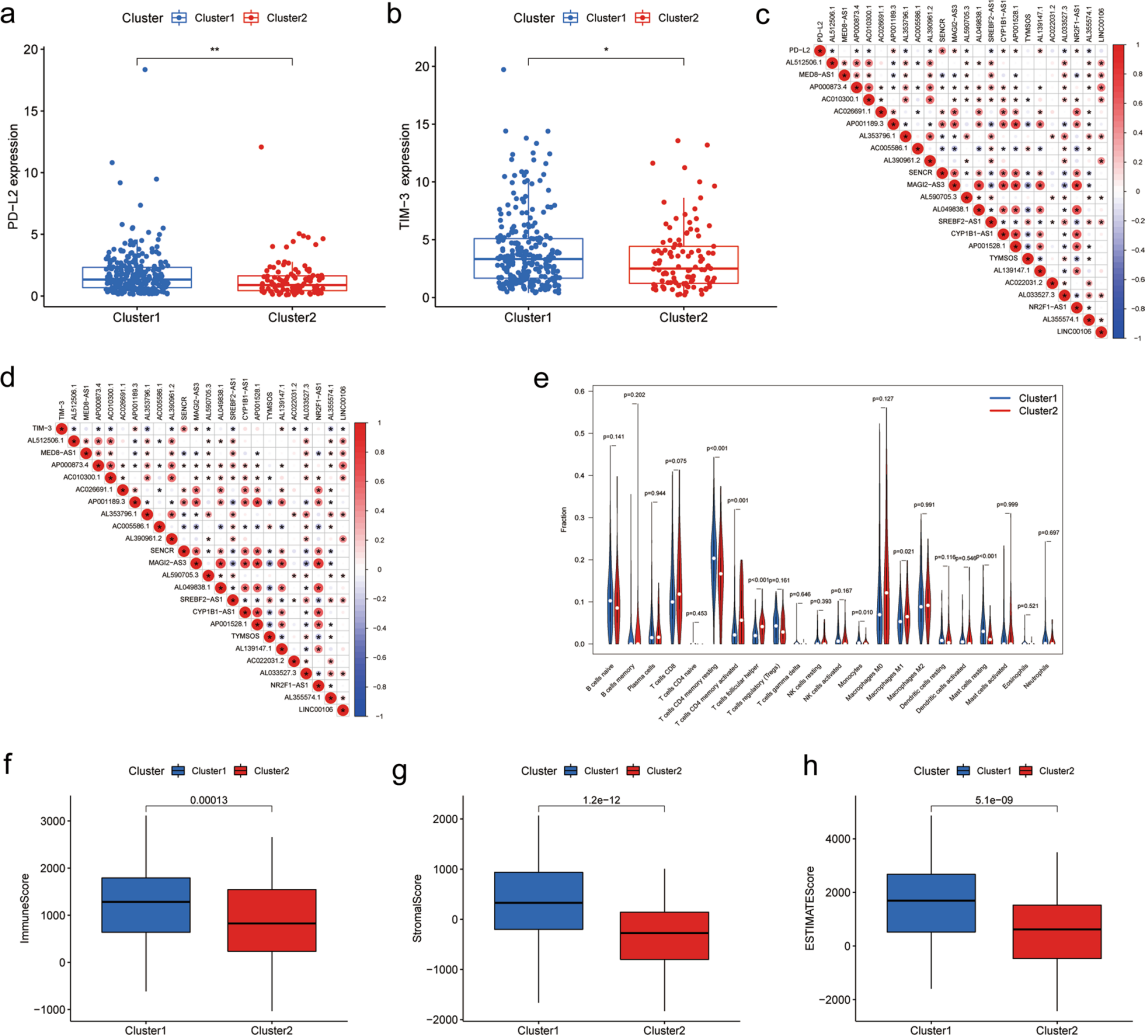
Immunity analysis of cluster1 and cluster2. **a**, **b** Differential expression analysis of immune checkpoints in cluster 1 and cluster 2: PD‐L2 (**a**) and TIM-3 (**b**). **c**, **d** Heatmap of the correlation among 23 m^6^A-related lncRNAs and PD‐L2 or TIM-3. The “*” represents the statistically significant P value (P < 0.05). Red color represents positive correlation, while blue color represents negative correlation. The depth of colors represents the correlation value, ranging from − 1 to 1. **e** The violin plot illustrating the different proportions of TICs in two clusters. **f–h** The box diagrams of immune scores, stromal scores, and ESTIMATE scores in two clusters. P < 0.05 *; and P < 0.01**. m^6^A, N6-methyladenosin; TIC, tumor-infiltrating immune cell

Given the fact that several immune-related signaling pathways were enriched in both clusters, we further made an analysis on immunity which was comprised of immune checkpoints expression, TIC abundance profile and TME scores, so as to investigate the difference between the two clusters. First of all, we analyzed differentially expressed immune checkpoints, including PD‐1, PD‐L1, PD‐L2, IDO1, CTLA‐4, TIM‐3, LAG3 and TIGIT. Compared to cluster2, expression levels of both PD‐L2 and TIM-3 were significantly upregulated in cluster1 (P < 0.05) (Fig. 4a, b).

Subsequently, the co-expression analysis between immune checkpoints and m^6^A-related prognostic lncRNAs was implemented in R software. Amid 23 m^6^A-related prognostic lncRNAs, 16 lncRNAs was significantly correlated with PD‐L2 (7 positive correlations and 9 negative correlations; P < 0.05) while 15 lncRNAs was closely relevant to TIM (4 positive correlations and 11 negative correlations; P < 0.05) (Fig. 4c, d). In comparison to cluster2, cluster1 had more mast cells resting (P = 0.0003), monocytes (P = 0.0096) and T cells CD4 memory resting (P = 0.00044), but less macrophages M1 (P = 0.021), T cells CD4 memory activated (P = 0.0012) and T cells follicular helper (P = 1.1e-05) amidst the TICs with differential profiles (Fig. 4e, Additional file 5: Figure S2). Besides, Immune, stromal, and ESTIMATE scores, being relevant to the ratio of the immune/stromal components, were higher in cluster1 than cluster2 (P < 0.05) (Fig. 4f–h). Altogether, these results suggested that m^6^A-related prognostic lncRNAs were intimately associated with tumor immunity.

### Construction of the m^6^A-LPS

**Fig. 5:**
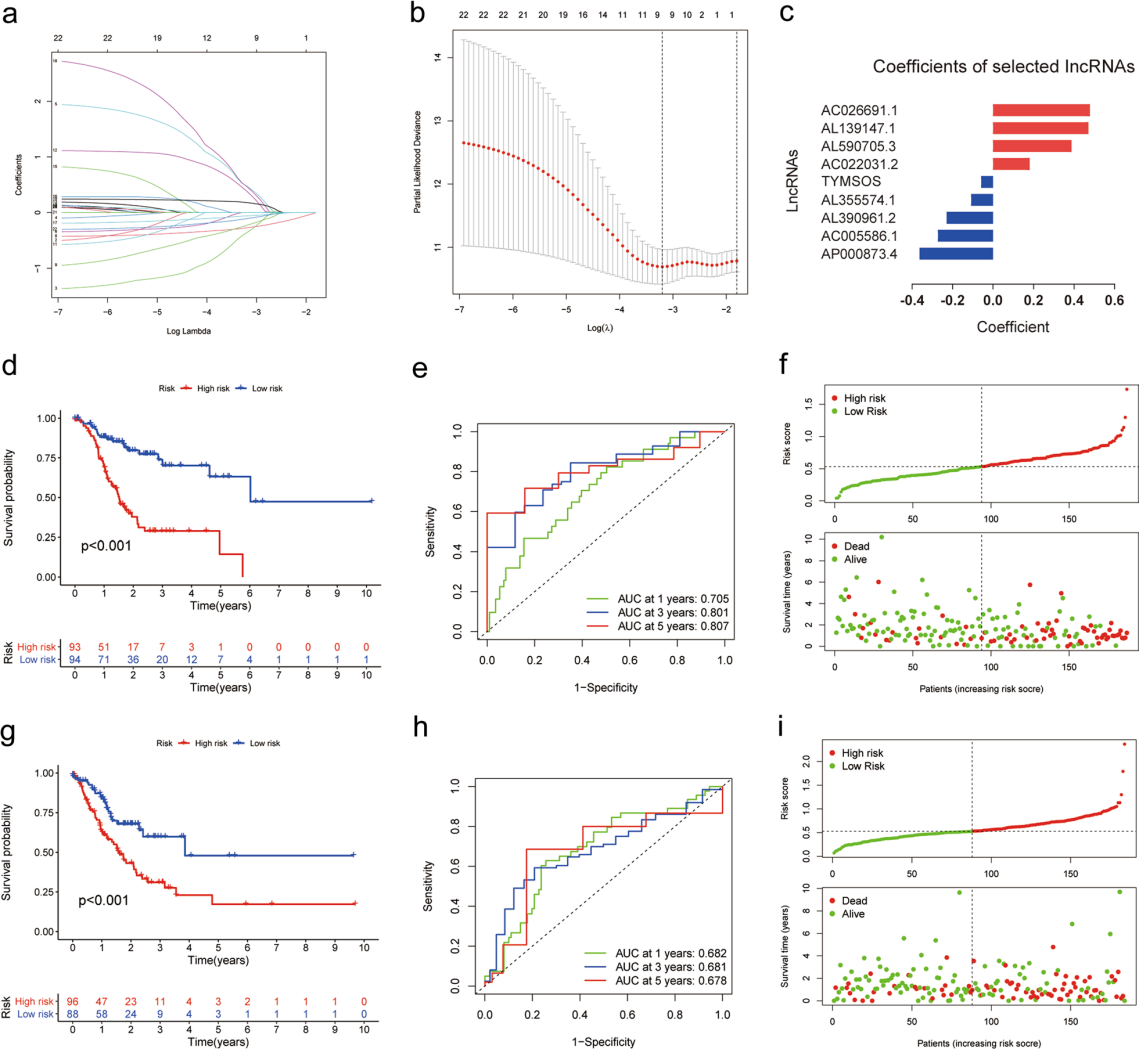
Construction and validation of the m^6^A-LPS. **a** The partial likelihood deviance plot. **b** Lasso coefficient profiles. **c** Coefficient of nine screened m^6^A-related lncRNAs in the Lasso model. **d**, **g** The K-M curve showed that the high-risk group had a more inferior OS than the low-risk group in train (**d**) and test sets (**g**). **e**, **h** ROC curve of the m^6^A-LPS: The AUCs of 1-, 3-, and 5-year OS in the train set (**e**) and test set (**h**). **f**, **i** Distributions of risk scores and survival status of GC patients in the train (**f**) and test sets (**i**). m^6^A-LPS, m^6^A-related lncRNA prognostic signature; Lasso, Least absolute shrinkage and selection operator; K-M, Kaplan–Meier; OS, overall survival; ROC, receiver operating characteristic; AUC, areas under the curve; GC, Gastric cancer

To investigate the prognostic role of m^6^A-related lncRNAs in GC, we employed the Lasso algorithm to construct a m^6^A-LPS and further combined it with 23 m^6^A-related prognostic lncRNAs obtained from previous univariate Cox regression analysis. Based on the optimal value of λ (λ = 9), we ultimately screened out nine m^6^A-related prognostic lncRNAs (P < 0.05) (Fig. 5a, b). Taking advantage of both regression coefficients and expression levels of nine m^6^A-related prognostic lncRNAs (AC026691.1, Coefficient = 0.4785; AL139147.1, Coefficient = 0.4706; AL590705.3, Coefficient = 0.3874; TYMSOS, Coefficient = -0.0586; AL355574.1, Coefficient = -0.1085; AL390961.2, Coefficient = -0.2289; AC005586.1, Coefficient = -0.2724; and AP000873.4, Coefficient = -0.3635), we estimated the risk score of the m^6^A-LPS (Fig. 5c; Additional file 6: Table S4).

In order to appraise the prognostic role of m^6^A-LPS, we divided GC patients into train and test sets randomly (Additional file 7: Table S5). Furthermore, based on the median value of the risk score, we stratified GC patients into high- and low-risk groups. K-M survival curves demonstrated that GC patients in the high‐risk group had lower OS rates than their counterparts in both train and test sets (P < 0.001) (Fig. 5d, g). Afterwards, the ROC curves were plotted to evaluate the accuracy of m^6^A-LPS in predicting survival of GC at 1, 3, and 5 years. (Train set: AUC at 1, 3, 5 year is 0.705, 0.801, and 0.807, respectively; Test set: 0.682, 0.681 and 0.678, separately; Fig. 5e, h). Besides, we assessed risk scores of each GC case amidst train and test sets, which implied that GC patients in the low‐risk group had better survival status and shorter dead status than high-risk group (Fig. 5f, i). In general, above results indicated that m^6^A-LPS had a promising capacity to predict the survival of GC patients.

### Independent prognostic analysis and stratification analysis

To determine whether the m^6^A-LPS could function as an independent prognostic indicator, we performed both univariate and multivariate Cox analysis on signature-based risk scores in train and test sets. The outcome of univariate Cox analysis suggested that m^6^A-LPS-based risk score was closely associated with unfavorable OS in both train set [hazard ratio (HR): 10.409, 95% CI 5.025–21.564, P < 0.001, Additional file 8: Figure S3a] and test set (HR: 4.490, 95% CI 1.823 − 11.061, P < 0.001, Additional file 8: Figure S3c). Moreover, the result of multivariate Cox regression analysis also demonstrated that corresponding risk score was intimately related to low OS in both train set (HR: 11.097, 95% CI 4.830 − 25.493, P = 0.001, Additional file 8: Figure S3b) and test set (HR: 6.411, 95% CI 2.394 − 17.172, P < 0.001, Additional file 8: Figure S3d). Meanwhile, age and stage were also verified to be closely relevant with the OS in univariate and multivariate Cox analysis. Generally speaking, these results confirmed that our m^6^A-LPS-based risk score might be an independent factor for prognostic evaluation.

**Fig. 6:**
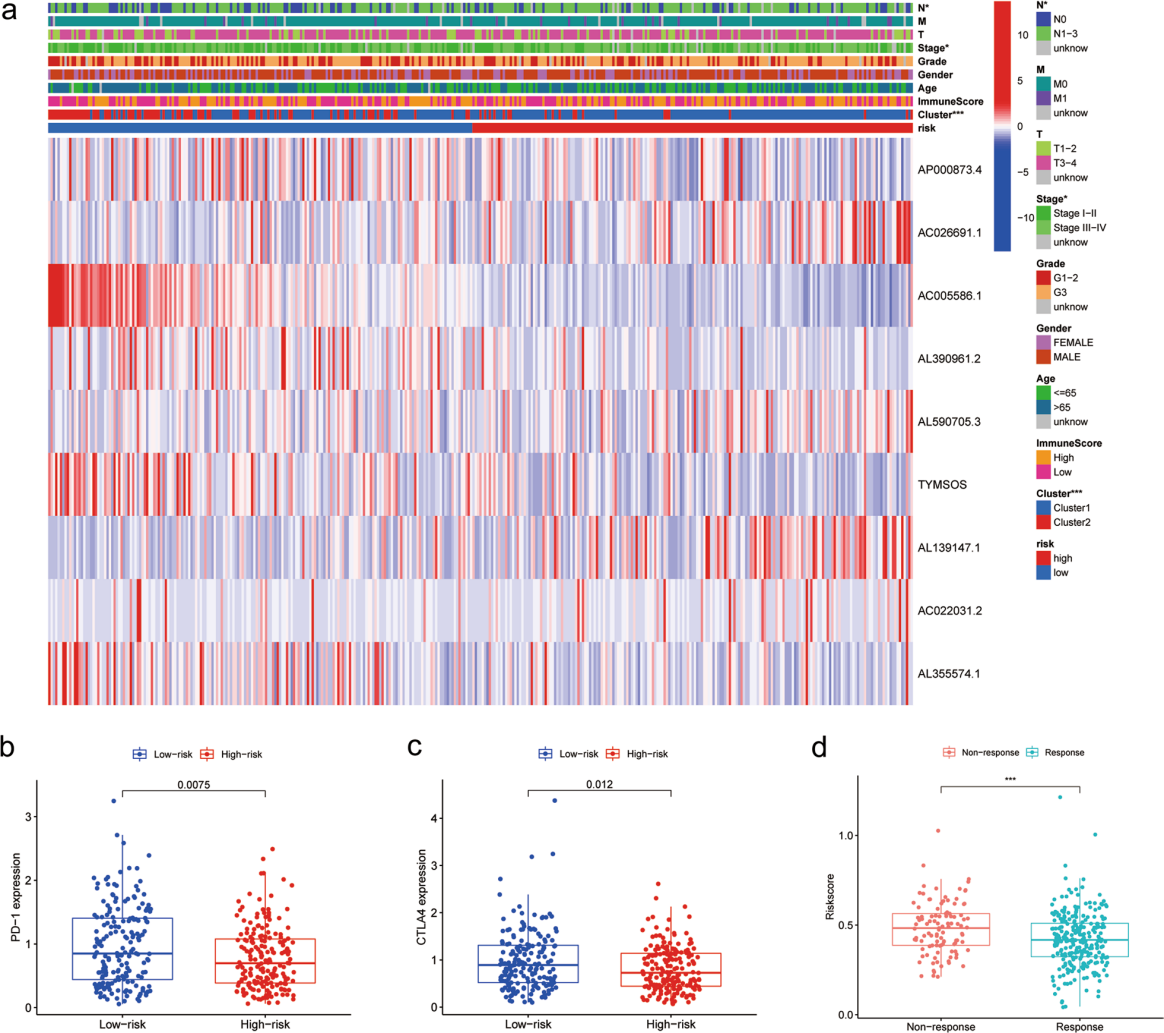
Correlation of the risk score and ICB therapy response. **a** The correlation analysis of the m^6^A-related lncRNAs, risk score and clinical factors. **b**, **c** The differential expression analysis of two essential immune checkpoints (PD-1 and CTLA4) in low-risk and high-risk groups. **d** The relationship between risk score and response to immunotherapy. m^6^A-LPS, m^6^A-related lncRNA prognostic signature; ICB, immune checkpoint blocker

Additionally, based on clinicopathological parameters containing age, gender, tumor stage and tumor grade, we employed stratification analysis to examine the predictive value on the survival of m^6^A-LPS in each section. The results demonstrated that the high-risk group had an intimate correlation with worse survival (P < 0.001) in both older and (≥ 65 years) younger group (< 65 years), male and female, advanced- (Stage III–IV) and early‐stage (Stage I–II) patients and tumor grade (G1–2 or G3) (Additional file 8: Figure S3e–l). The outcomes suggested that the m^6^A-LPS was adept in stably discriminating patients with undesirable prognosis. Besides, the overall correlation between risk score and clinical factors was plotted in Fig. 6a.

### Correlation between the risk score and ICB therapy response

To further explore the underlying immune-related mechanism in GC patients, the differential expression of immune checkpoints analysis and TICs risk score were identified. Intriguingly, we found that expression levels of PD-1 and CTLA4 in the low-risk group were higher than in the high-risk one (Fig. 6b, c), which indicated that there were more immune escape and more protein expression of immune checkpoints in the low-risk group. On account of above findings, we further investigated the role of these lncRNAs in ICBs therapy. It was implied that the patients who responded to ICBs therapy had a lower risk score than patients who did not (Fig. 6d), suggesting that low-risk patients were underlying beneficiaries of ICBs therapy. Furthermore, we found that the risk score had significant negative correlations (P < 0.05) with B cells memory (R =  − 0.18), Macrophages M0 (R =  − 0.18), T cells CD4 memory activated (R =  − 0.23), and T cells follicular helper (R =  − 0.28). Meanwhile, the risk score was positively related to DCs resting (R = 0.21), Macrophages M2 (R = 0.28), Mast cells resting (R = 0.22), NK cells activated (R = 0.15), T cells CD4 memory resting (R = 0.24), and Monocytes (R = 0.31) (Additional file 9: Figure S4).

### Validation of the m^6^A-LPS in GC tissues

We collected ten pairs of tumor tissues and para tumor tissues from Xijing Hospital. Via performing qRT-PCR, we found that two m^6^A-related lncRNAs (AC022031.2 and AL590705.3) were upregulated, while seven lncRNAs (TYMSOS, AC026691.1, AL355574.1, AP000873.4, AL390961.2, AC005586.1, and AL139147.1) were downregulated in cancer tissues (Additional file 10: Figure S5).

### Reduced lncRNA AC026691.1 could promote proliferation and migration of GC cells

**Fig. 7:**
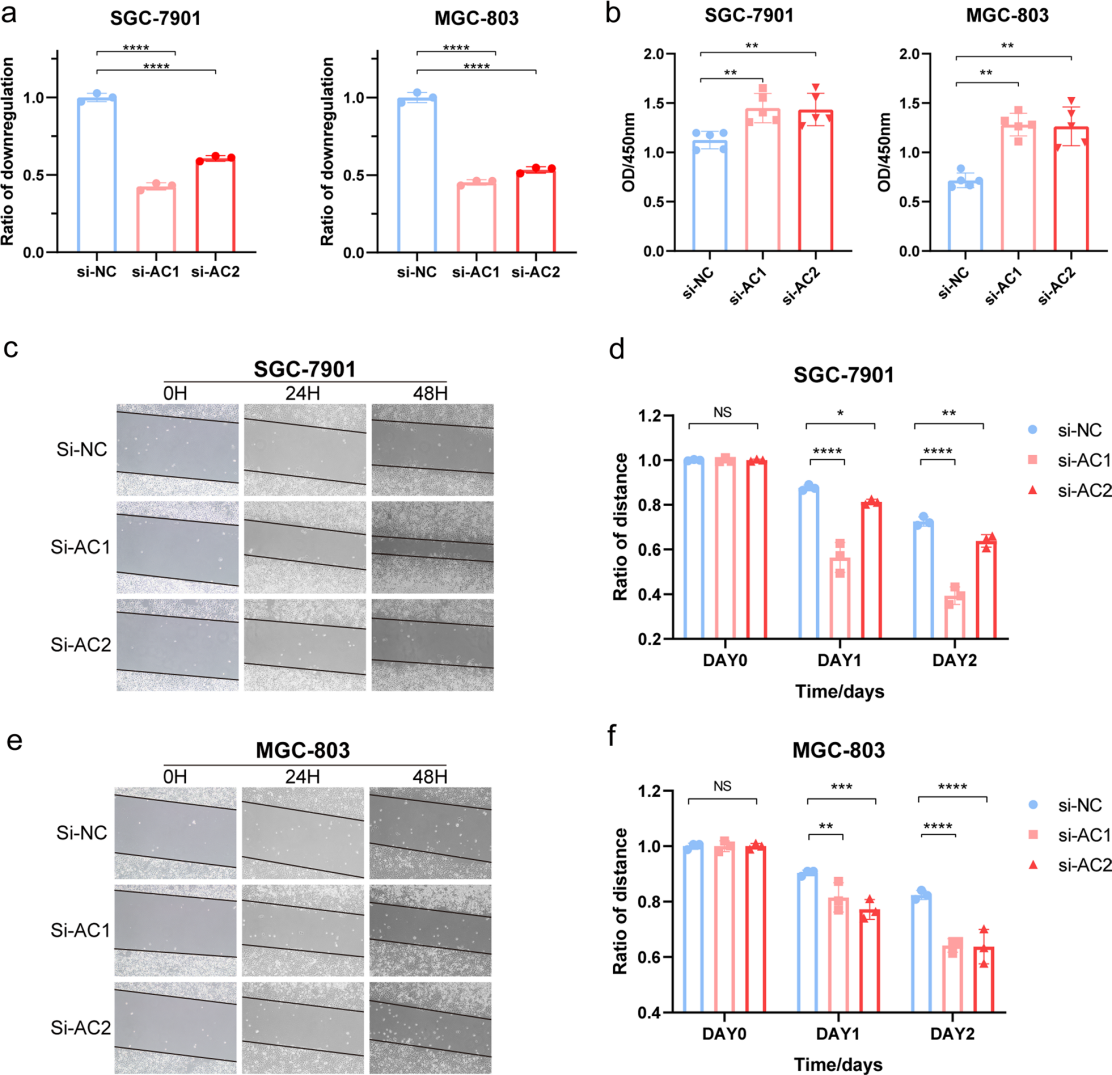
Downregulated lncRNA AC026691.1 promoted cell proliferation and migration of GC cell lines. **a** LncRNA AC026691.1 knockdown efficiency in SGC-7901 and BGC-803 cell lines.** b** CCK-8 assay to estimate the ability of proliferation in GC cell lines. **c–f** Scrape wound-healing assay to evaluate the ability of migration in two GC cell lines. P < 0.05 *; P < 0.01**; P < 0.001***; and P < 0.0001****. lncRNA, long noncoding RNA; GC, Gastric cancer; CCK-8, Cell Counting Kit-8

In view of the above findings, lncRNA AC026691.1 had the highest regression coefficients. To validate the tumorigenic role m^6^A-related lncRNA played in GC, siRNA was employed to silence the lncRNA AC026691.1 in SGC-7901 and BGC-803 cells. Moreover, we detected the transfection efficiency with qRT-PCR (Fig. 7a). As indicated in the results of CCK-8 assay, via silencing with siRNA, the ability of cell proliferation was dramatically enhanced in SGC-7901 and BGC-803 cells (Fig. 7b). Additionally, outcomes of the wound-healing assay demonstrated that the migration capability was significantly promoted (Fig. 7c–f). Taken above results together, it was natural to summarize that lncRNA AC026691.1 could play a vital role in suppressing GC.

### lncRNA AC026691.1 had a strong interaction with FTO in m^6^A modification of GC

**Fig. 8:**
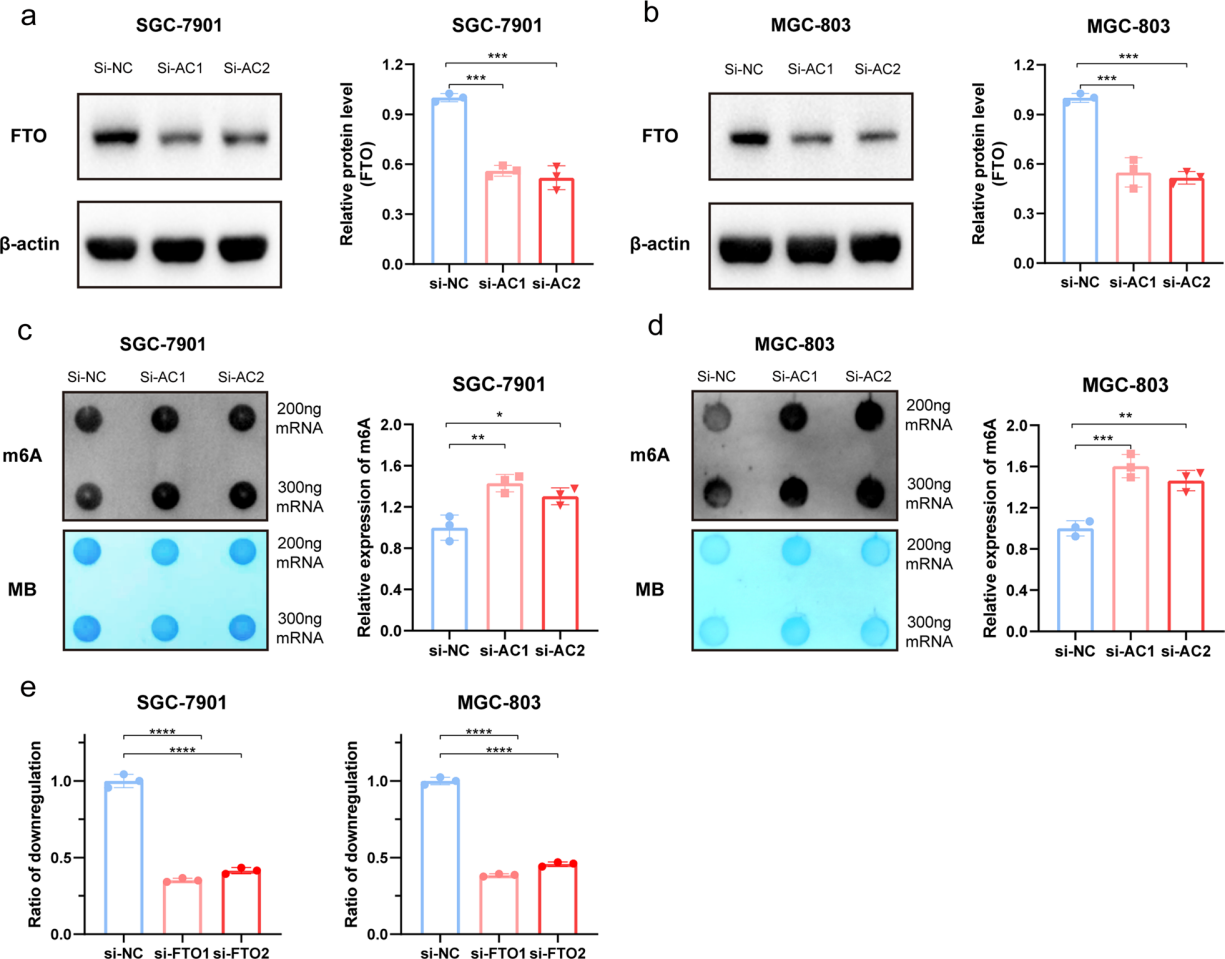
The interaction of lncRNA AC026691.1 and *FTO* in GC cell lines. **a** Relative FTO expression levels in SGC-7901 and BGC-803 cell lines which were silenced by siRNA of AC026691.1. **b** Dot blot to detect the m^6^A levels in two GC cell lines after silencing the lncRNA AC026691.1. **c** Relative downregulation levels of AC026691.1 in GC cells which were silenced by si-FTO. P < 0.05 *; P < 0.01**; P < 0.001***; and P < 0.0001****. lncRNA, long noncoding RNA; FTO, fat mass and obesity-associated protein; GC, Gastric cancer

To explore the underlying mechanism of lncRNA AC026691.1 in m^6^A modification, we made correlation analysis between AC026691.1 and m^6^A-related genes. Corresponding result suggested that lncRNA AC026691.1 had the most positive relation with *FTO* (Coefficient = 0.5080, P = 5.43e−26) (Additional file 11: Table S6). After silencing lncRNA AC026691.1, the expression level of FTO comparatively decreased in SGC-7901 and BGC-803 cells (P < 0.05) (Fig. 8a, b). Additionally, the m^6^A level exhibited a downward tendency in GC cell lines after downregulating the lncRNA AC026691.1 (P < 0.05) (Fig. 8c, d). Furthermore, by downregulation of *FTO* in GC cell lines, the expression level of LncRNA AC026691.1 dramatically declined in qRT-PCR results (P < 0.05) (Fig. 8e). And the underlying regulatory site of *FTO* was further predicted and demonstrated in Additional file 12: Figure S6. In general, the above results indicated that the lncRNA AC026691.1 interacted closely with *FTO*, via regulation of which, the expression level of m^6^A got reduced.

## Discussion

GC is universally acknowledged as one of the most prevalent gastrointestinal tract malignancies with considerably high morbidity and mortality, and an increasing quantity of attention is paid to GC annually [2]. m^6^A is identified as the most prominent and abundant post-transcriptional modification in eukaryotic RNAs. In addition, m^6^A is also widely recognized to play a critical role in multitype tumors through various mechanisms [6, 26]. Scientists have recently validated the nonnegligible significance of m^6^A regulator-mediated methylation modification in GC [27]. As a category of tumor biomarker, LncRNA has drawn increasing attention in the field of early screening, targeted therapy and prognostic evaluation [28]. In GC, Multitudes of reports have verified that lncRNA is of undeniable importance in neoplastic invasiveness and clinical prognosis [29]. Convincing evidence indicated that lncRNAs play vital roles in the immune system, especially in cancer immunity. On the one hand, lncRNAs could regulate both differentiation and function of immune cells. On the other hand, lncRNAs could affect proliferation, differentiation, infiltration, and metastasis of cancer cells [30]. Besides, plentiful researches suggested that m^6^A could modify lncRNAs, which contributed to tumorigenesis of multitype cancers including proliferation, invasion, and metastasis [31].Integrating above-mentioned evidence, we were confident that m^6^A modification targeted at lncRNAs could affect both onset and progression of GC. Nevertheless, whether and how m^6^A-modified-lncRNAs function in GC and immunity are still not entirely known currently.

In our present study, we acquired 32 paracancerous and 375 cancerous GC samples from TCGA dataset and constructed a m^6^A-LPS based on nine most significant m^6^A-related prognostic lncRNAs. The immunity-related analysis demonstrated that m^6^A modification of lncRNAs might negatively modulate the expression of immune checkpoint (PD-1 and CTLA4). Besides, expression level of the most vital lncRNA in m^6^A-LPS (lncRNA AC026691.1) was observed to decline in clinical samples of GC. Migration and proliferation experiments further confirmed the negative regulating role that lncRNA AC026691.1 played in GC cell lines. Afterwards, the result of co-expression analysis indicated that *FTO* had a highly positive correlation with AC026691.1, which was further verified by qRT-PCR. Moreover, after silencing the lncRNA AC026691.1, expression levels of FTO and m^6^A downregulated in GC cell lines. Generally speaking, we came to the following conclusion that lncRNA AC026691.1 and *FTO* were intimately associated in the regulation of m^6^A RNA methyladenine in GC. In addition, combined effect of lncRNA AC026691.1 and *FTO* might suppress GC via downregulation of m^6^A level, being a novel therapeutic target of GC.

Our results screened nine hub m^6^A-related lncRNAs and built a superb model to accurately predict the clinical outcomes of GC patients. Among the m^6^A-related lncRNAs, we discovered a novel protective lncRNA AC026691.1, which was relatively low expressed in tumor samples. lncRNA AC026691.1could inhibit both proliferative and migrating abilities of GC. In light that multitudes of tumor-related lncRNAs were reported, lncRNA has been recognized to play an irreplaceable role in GC. A previous study has verified that lncRNA MEG3 could serve as a tumor suppressor to inhibit both proliferation and metastasis of GC [32]. Besides, lncRNA HOXA cluster antisense RNA2 was found to express aberrantly in plentiful malignancies including GC [33]. Unlike above-mentioned investigations, lncRNA AC026691.1 was first reported in GC and the underlying mechanisms of lncRNA AC026691.1 in other tumors left a wide scope for further research.

Considering above result of the correlation analysis, *FTO* was a m^6^A-related gene that was most relevant to the lncRNA AC026691.1. Our current study indicated that *FTO* and lncRNA AC026691.1 have an intimate interaction with m^6^A RNA methyladenine process in GC. FTO is a significant m^6^A demethylase, which plays a critical role in the most common modified nucleoside [34]. Moreover, FTO is verified to be widely involved in various tumorigenesis by m6A-dependent demethylase activity. Researchers found that FTO could effectively promote cell proliferation, colony formation, and metastatic process in breast cancer [35]. And FTO facilitated malignant phenotypes of lung squamous cells such as proliferation and invasiveness, and inhibited cell apoptosis [36]. Unlike other tumors, emerging evidence has demonstrated that *FTO* is associated with inhibition of tumor progression in GC. Published reports also indicated that knockdown of *FTO* could upregulate the expression level of m^6^A, which further reinforced both proliferation and invasion of GC via activating Wnt and PI3K-Akt signaling pathways [37]. Besides, a recent study has further implied the expression level of FTO was significantly downregulated both in vitro and in vivo [38].

Interestingly, we found that the low-risk group demonstrated a relatively higher expression level of immune checkpoint PD-1 and CTLA4 and was more responsive to immunotherapy. ICBs are universally deemed as a novel therapeutic strategy, especially for chemorefractory GC. Compelling evidence has indicated that application of anti-PD-1 therapy to GC patients could apparently prolong their OS in earlier lines of treatment [39]. Pitifully, both clinical study and application of anti-CTLA4 antibodies were merely confined to metastatic melanoma [40, 41]. According to our findings, CTLA4 inhibitor could be a potential research direction for targeted immunotherapy in GC. Additionally, given the superior capability of our m^6^A-LPS in predicting therapeutic effect, it might provide considerable value for better application of immunotherapy.

Nevertheless, there were several limitations in our current study. First and foremost, our research merely based on a publicly available dataset. More prospective real-world data ought to be incorporated in our research so as to validate the clinical utility of our established model. In addition, apart from in vitro experiments, more in vivo one should be made to comprehensively explore regulatory mechanisms of these lncRNAs.

## Conclusion

Collectively, we have offered novel insights into functions of m^6^A-related lncRNA and first constructed a brand new prognosis-related lncRNA signature with high predictive value in GC. For the first time, we elucidated that m^6^A-related lncRNAs might play indispensable roles in TICs and influence the anti-cancer ability of ICBs. To summarize, the m^6^A-related lncRNA could potentially act as an indicator for the response to immunotherapy. We also found that *FTO*-regulated AC026691.1 might function as an essential tumor suppression lncRNA in GC.

## Supplementary Information

**Additional file 1: Table S1.** SiRNA sequence for lncRNA AC026691.1 and FTO.

**Additional file 2: Table S2.** Primers for qRT-PCR in our study.

**Additional file 3: Table S3.** The twenty-three m6A-related prognostic lncRNAs.

**Additional file 4: Figure S1.** Functional enrichment analysis of cluster1 and cluster2. **(a, b)** Enriched tumor hallmarks in cluster2: cell cycle and P53 signaling pathway. **(c, d)** Enriched tumor hallmarks in cluster1: ECM receptor interaction and MAPK signaling pathway. **(e–h)** Several significant immunologic characteristics of cluster1 and cluster2.

**Additional file 5: Figure S2.** TICs with differential profiles between cluster1 and cluster2**. (a)** Macrophages M1, **(b)** mast cells resting, **(c)** monocytes, **(d)** T cells CD4 memory activated, **(e)** T cells CD4 memory resting, **(f)** T cells follicular helper. TIC, tumor-infiltrating immune cell.

**Additional file 6: Table S4.** Coefficient of LASSO model in this study.

**Additional file 7: Table S5.** Baseline of training and testing sets.

**Additional file 8: Figure S3.** Independent prognosis and stratification analysis of the m^6^A‐LPS. **(a, b)** Univariate analysis and Multivariate analysis of the lncRNA model in the train set. **(c, d)** Univariate analysis and Multivariate analysis in the test set. **(e–l)** The survival of the m^6^A‐LPS for GC stratified by age, gender, tumor stage and tumor grade. m^6^A-LPS, m^6^A-related lncRNA prognostic signature; GC, Gastric cancer.

**Additional file 9: Figure S4.** Association between the m^6^A-LPS and immune cells. **(a)** B cells memory, **(b)** macrophages M0, **(c)** T cells CD4 memory activated, **(d)** T cells follicular helper, **(e)** DCs resting, **(f)** macrophages M2, **(g)** mast cells resting, **(h)** NK cells activated, **(i)** T cells CD4 memory resting, and **(j)** Monocytes. m^6^A-LPS, m^6^A-related lncRNA prognostic signature.

**Additional file 10: Figure S5.** Expression of m^6^A-related lncRNAs in GC patients. Relative of RNA expression of lncRNAs between cancerous and adjacent normal tissues: **(a)** TYMSOS, **(b)** AC022031.2, **(c)** AL355574.1, **(d)** AP000873.4, **(e)** AL590705.3, **(f)** AL390961.2, **(g)** AC026691.1, **(h)** AC005586.1, and **(i)** AL139147.1. P < 0.05 * and P < 0.01 **.

**Additional file 11: Table S6.** Correlation of LncRNA AC026691.1 and m6A-related genes.

**Additional file 12: Figure S6.** Potential m^6^A modification positions of lncRNA AC026691.1. **(a)** The underlying modification sites distributed along the sequence of lncRNA AC026691.1. **(b)** The detailed information about prediction positions. m^6^A, N6-methyladenosin; lncRNA, long noncoding RNA.

## Data Availability

The datasets used and/or analyzed during the current study are included in this published article and its additional files.

## Abbreviations

GC: Gastric cancer
m^6^A: N6-methyladenosine
lncRNA: Long noncoding RNA
m^6^A-LPS: M^6^A-related lncRNA prognostic signature
MALAT1: Metastasis-associated lung adenocarcinoma transcript 1
TME: Tumor microenvironment
ICB: Immune checkpoint blocker
FTO: Fat mass and obesity-associated protein
TCGA: The Cancer Genome Atlas
K-M: Kaplan–Meier
GSEA: Gene set enrichment analysis
TIC: Tumor-infiltrating immune cell
Lasso: Least absolute shrinkage and selection operator
AUC: Areas under the curve
ROC: Receiver operating characteristic
PD‐1: Programmed death 1
PD‐L1: Programmed death ligand 1
PD‐L2: Programmed death ligand 2
IDO1: Indoleamine 2,3-dioxygenase 1
CTLA‐4: Cytotoxic T‐lymphocyte antigen 4
TIM‐3: T‐cell immunoglobulin domain, mucin domain‐containing molecule‐3
LAG3: Lymphocyte-activation gene 3
TIGIT: T cell immunoreceptor with Ig and ITIM domains
siRNA: Small interference RNA
CCK-8: Cell Counting Kit-8
qRT-PCR: Quantitative real-time polymerase chain reaction
DC: Dendritic cells
NK: Natural Killer
OS: Overall survival
HR: Hazard ratio
