# Supplementary material for: Identification of a robust signature for clinical outcomes and immunotherapy response in gastric cancer: based on N6-methyladenosine related long noncoding RNAs
Source: Cancer Cell Int. 2021 Aug 16;21:432. doi: 10.1186/s12935-021-02146-w (PMC8365962; doi:10.1186/s12935-021-02146-w)
Supplement: Supplementary file 1 — Additional file 1: Table S1. SiRNA sequence for lncRNA AC026691.1 and FTO. [file 12935_2021_2146_MOESM1_ESM.docx]

**Table S1.** SiRNA sequence for lncRNA AC026691.1 and FTO.

| **SiRNA** | **Sequence** |
| --- | --- |
| AC026691.1-1 | sense (5'-3'): GGUUGAUGUGAAGUUUAAATT |
|  | antisense (5'-3'): UUUAAACUUCACAUCAACCTT |
| AC026691.1-2 | sense (5'-3'): GCUUUACACCUUACUUUAATT |
|  | antisense (5'-3'): UUAAAGUAAGGUGUAAAGCTT |
| FTO-1 | sense (5'-3'): GAUUCUAGUUACAGACUUATT |
|  | antisense (5'-3'): UAAGUCUGUAACUAGAAUCTT |
| FTO-2 | sense (5'-3'): GCUUGGUGUUAAAGAUUAATT |
|  | antisense (5'-3'): UUAAUCUUUAACACCAAGCTT |
