# Supplementary material for: Identification of a robust signature for clinical outcomes and immunotherapy response in gastric cancer: based on N6-methyladenosine related long noncoding RNAs
Source: Cancer Cell Int. 2021 Aug 16;21:432. doi: 10.1186/s12935-021-02146-w (PMC8365962; doi:10.1186/s12935-021-02146-w)
Supplement: Supplementary file 2 — Additional file 2: Table S2. Primers for qRT-PCR in our study. [file 12935_2021_2146_MOESM2_ESM.docx]

**Table s2. Primers for qRT-PCR in our study**

| **lncRNA** | **Primer sequence** |
| --- | --- |
| AP000873.4 | Forward 5'-TCGTGATCTGCCCACCTTG-3' |
|  | Reverse 5'-TTTTAAATTCTGGGCTCCCT-3' |
| AC026691.1 | Forward 5'-CTCACCGGGATGCTTTACACC-3' |
|  | Reverse 5'-ATCCAGCACCCAAATCGATG-3' |
| AC005586.1 | Forward 5'-ATGACTGTGCCTTCACTCTGC-3' |
|  | Reverse 5'-AAAGGCTCAGGAATCCCTTCG-3' |
| AL390961.2 | Forward 5'-ATTCAGGTGGCTTAAAACTCA-3' |
|  | Reverse 5'-AATTTCACTCTTATTGCCCAG-3' |
| AL590705.3 | Forward 5'-TTTAATTGTGGTTCTGCCAAG-3' |
|  | Reverse 5'-TAGTTTTGGTTACAGGCTCC-3' |
| TYMSOS | Forward 5'-TTGACCAACCTGATCGCCTCGT-3' |
|  | Reverse 5'-ACTGTCCTGATGTACCTGCTCT-3' |
| AL139147.1 | Forward 5'-TGCCAAGACAATCATAAGCAA-3' |
|  | Reverse 5'-TTTTCCTGTGCAGAAGCTC-3' |
| AC022031.2 | Forward 5'-GAAATCACCCGTCTTCTGC-3' |
|  | Reverse 5'-GATGTCTATCTCCAGCTTCCA-3' |
| AL355574.1 | Forward 5'-CCGTTGCTTTCCTCAGGCTCT-3' |
|  | Reverse 5'-TATGGCTTTCCTTGACCGCTCT-3' |
