# Supplementary material for: Identification of a robust signature for clinical outcomes and immunotherapy response in gastric cancer: based on N6-methyladenosine related long noncoding RNAs
Source: Cancer Cell Int. 2021 Aug 16;21:432. doi: 10.1186/s12935-021-02146-w (PMC8365962; doi:10.1186/s12935-021-02146-w)
Supplement: Supplementary file 3 — Additional file 3: Table S3. The twenty-three m6A-related prognostic lncRNAs. [file 12935_2021_2146_MOESM3_ESM.docx]

**Table S3. The twenty-three m6A-related prognostic lncRNAs.**

| **m6A-related lncRNA** | **HR** | **HR.95L** | **HR.95H** | **P value** |
| --- | --- | --- | --- | --- |
| AL512506.1 | 0.4839 | 0.2542 | 0.9213 | 0.0272 |
| MED8-AS1 | 0.5247 | 0.2836 | 0.9705 | 0.0399 |
| AP000873.4 | 0.4601 | 0.2370 | 0.8932 | 0.0218 |
| AC010300.1 | 0.3967 | 0.1780 | 0.8840 | 0.0237 |
| AC026691.1 | 2.3025 | 1.0285 | 5.1546 | 0.0425 |
| AP001189.3 | 1.1887 | 1.0343 | 1.3662 | 0.0149 |
| AL353796.1 | 0.6688 | 0.4606 | 0.9711 | 0.0345 |
| AC005586.1 | 0.7664 | 0.6493 | 0.9046 | 0.0017 |
| AL390961.2 | 0.4462 | 0.2230 | 0.8928 | 0.0226 |
| SENCR | 1.5682 | 1.0817 | 2.2735 | 0.0176 |
| MAGI2-AS3 | 1.2075 | 1.0231 | 1.4252 | 0.0258 |
| AL590705.3 | 2.0355 | 1.2202 | 3.3954 | 0.0065 |
| AL049838.1 | 1.3905 | 1.0331 | 1.8715 | 0.0296 |
| SREBF2-AS1 | 0.7572 | 0.5889 | 0.9736 | 0.0301 |
| CYP1B1-AS1 | 3.4954 | 1.1157 | 10.9512 | 0.0317 |
| AP001528.1 | 1.4243 | 1.1067 | 1.8331 | 0.0060 |
| TYMSOS | 0.9031 | 0.8188 | 0.9961 | 0.0414 |
| AL139147.1 | 3.7629 | 1.4985 | 9.4495 | 0.0048 |
| AC022031.2 | 1.2135 | 1.0209 | 1.4425 | 0.0282 |
| AL033527.3 | 0.2960 | 0.1095 | 0.8005 | 0.0165 |
| NR2F1-AS1 | 1.3372 | 1.0107 | 1.7691 | 0.0419 |
| AL355574.1 | 0.8098 | 0.6983 | 0.9391 | 0.0052 |
| LINC00106 | 0.8707 | 0.7690 | 0.9858 | 0.0288 |
