# Supplementary figures and images for: Identification of a robust signature for clinical outcomes and immunotherapy response in gastric cancer: based on N6-methyladenosine related long noncoding RNAs
Source: Cancer Cell Int. 2021 Aug 16;21:432. doi: 10.1186/s12935-021-02146-w (PMC8365962; doi:10.1186/s12935-021-02146-w)

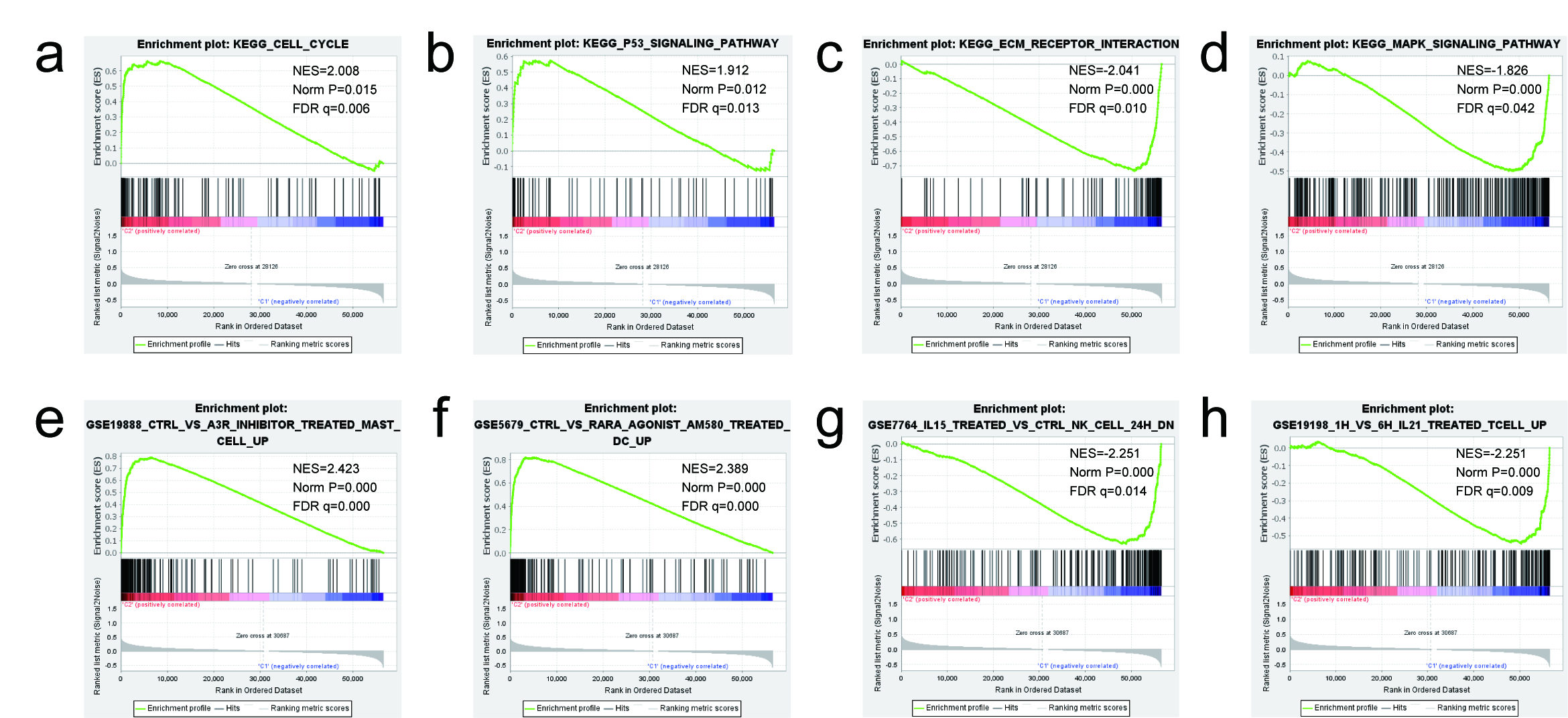

Supplement: Supplementary file 4 — Additional file 4: Figure S1. Functional enrichment analysis of cluster1 and cluster2. (a, b) Enriched tumor hallmarks in cluster2: cell cycle and P53 signaling pathway. (c, d) Enriched tumor hallmarks in cluster1: ECM receptor interaction and MAPK signaling pathway. (e–h) Several significant immunologic characteristics of cluster1 and cluster2. [file 12935_2021_2146_MOESM4_ESM.tif]

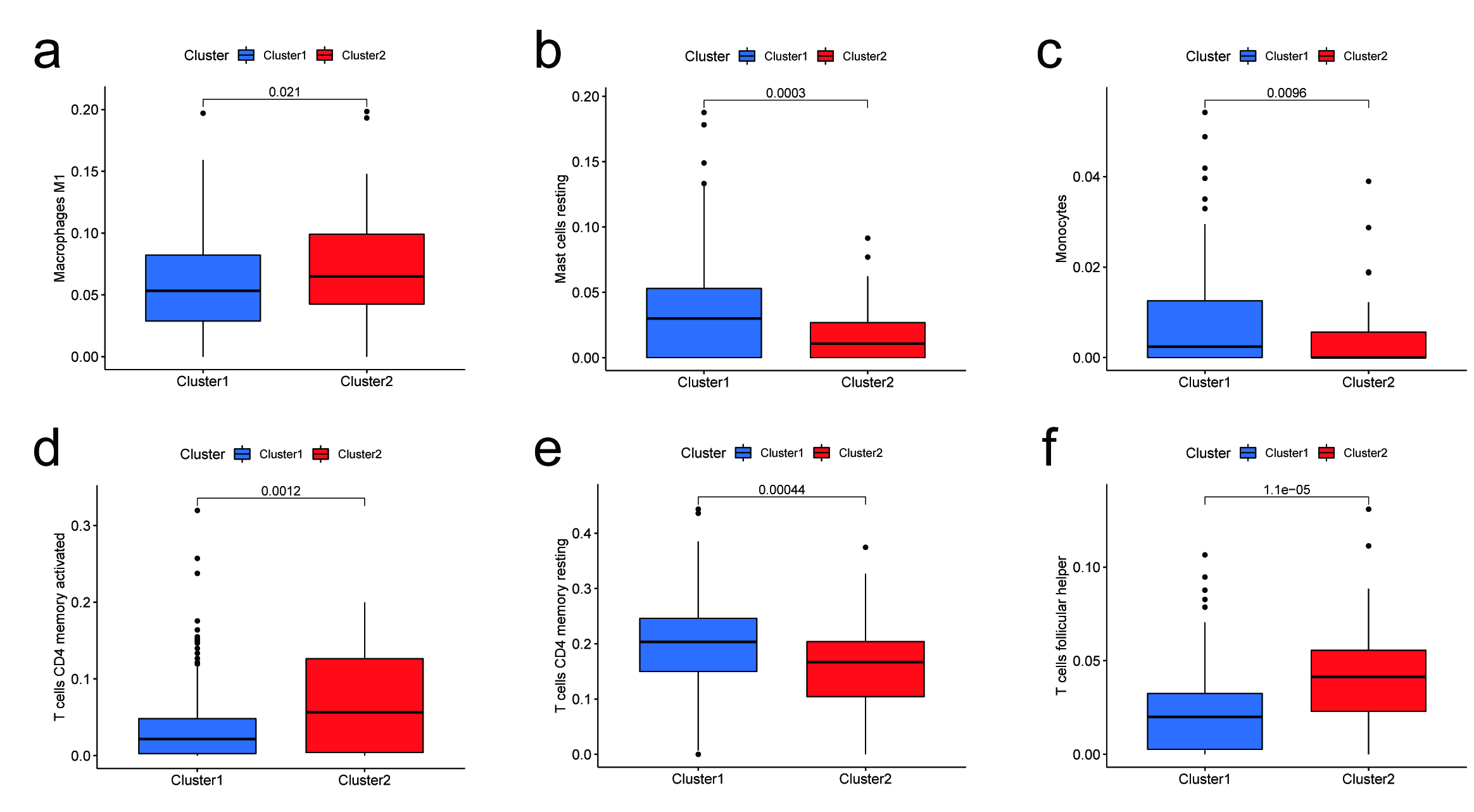

Supplement: Supplementary file 5 — Additional file 5: Figure S2. TICs with differential profiles between cluster1 and cluster2. (a) Macrophages M1, (b) mast cells resting, (c) monocytes, (d) T cells CD4 memory activated, (e) T cells CD4 memory resting, (f) T cells follicular helper. TIC, tumor-infiltrating immune cell. [file 12935_2021_2146_MOESM5_ESM.tif]

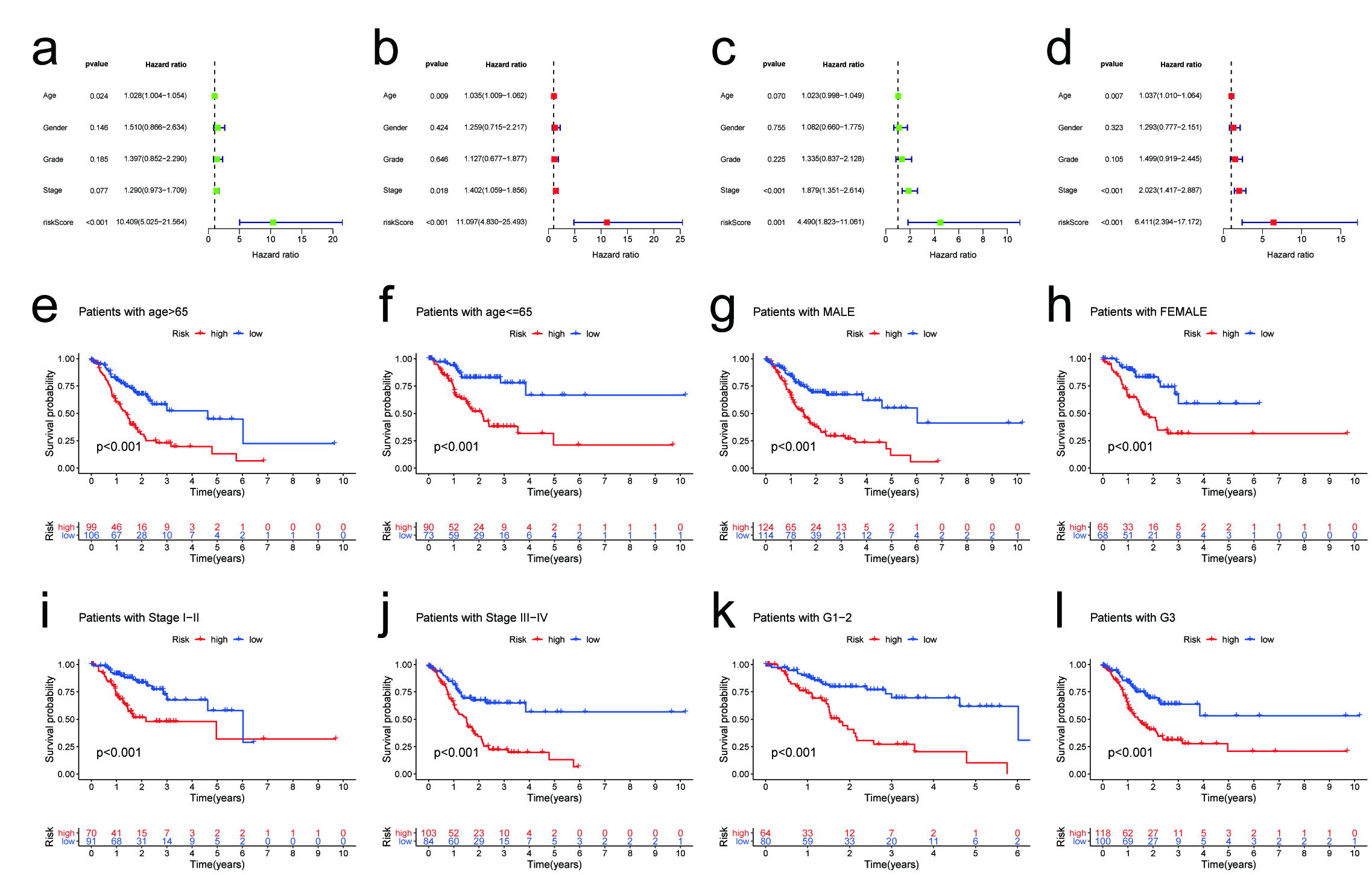

Supplement: Supplementary file 8 — Additional file 8: Figure S3. Independent prognosis and stratification analysis of the m6A‐LPS. (a, b) Univariate analysis and Multivariate analysis of the lncRNA model in the train set. (c, d) Univariate analysis and Multivariate analysis in the test set. (e–l) The survival of the m6A‐LPS for GC stratified by age, gender, tumor stage and tumor grade. m6A-LPS, m6A-related lncRNA prognostic signature; GC, Gastric cancer. [file 12935_2021_2146_MOESM8_ESM.tif]

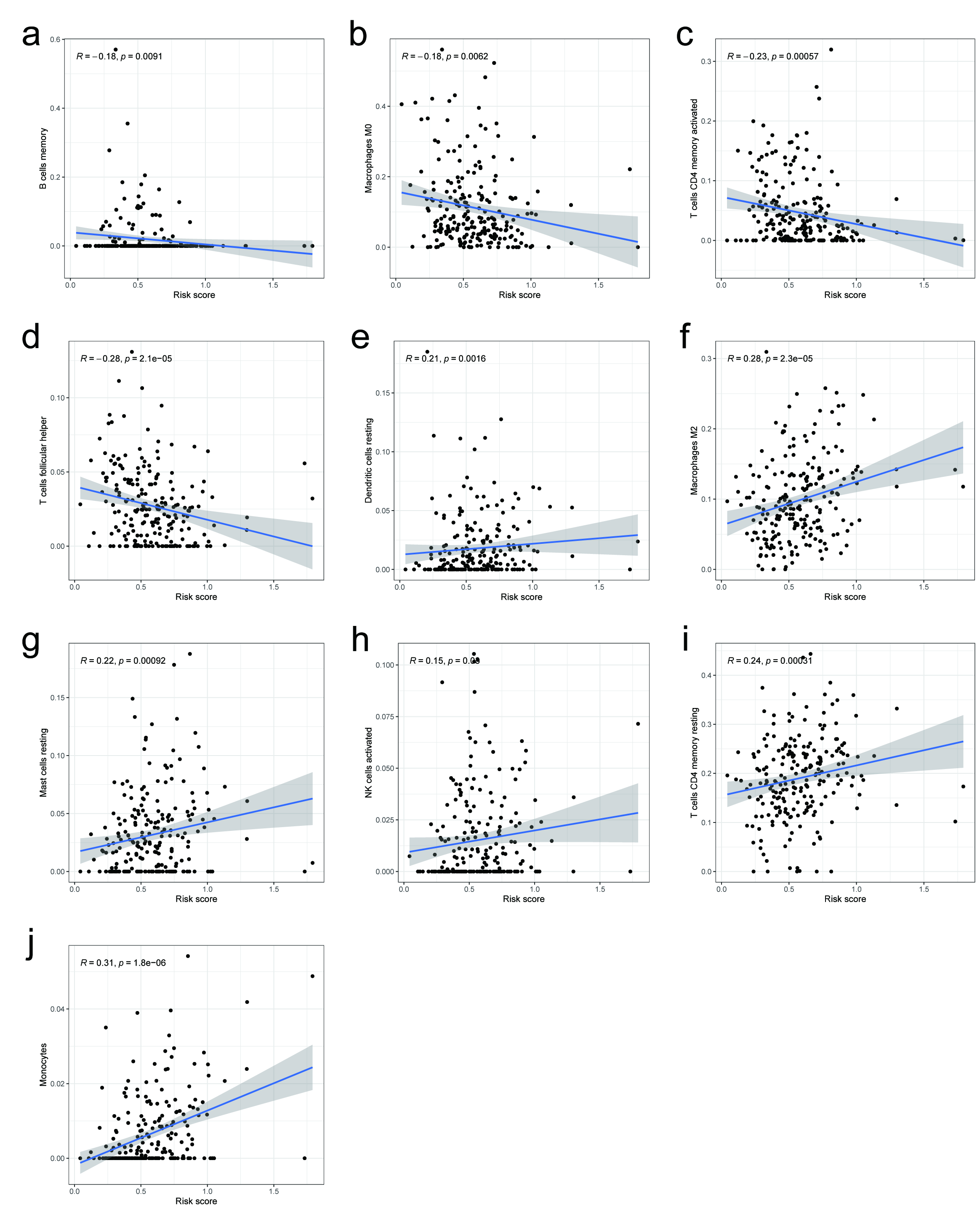

Supplement: Supplementary file 9 — Additional file 9: Figure S4. Association between the m6A-LPS and immune cells. (a) B cells memory, (b) macrophages M0, (c) T cells CD4 memory activated, (d) T cells follicular helper, (e) DCs resting, (f) macrophages M2, (g) mast cells resting, (h) NK cells activated, (i) T cells CD4 memory resting, and (j) Monocytes. m6A-LPS, m6A-related lncRNA prognostic signature. [file 12935_2021_2146_MOESM9_ESM.tif]

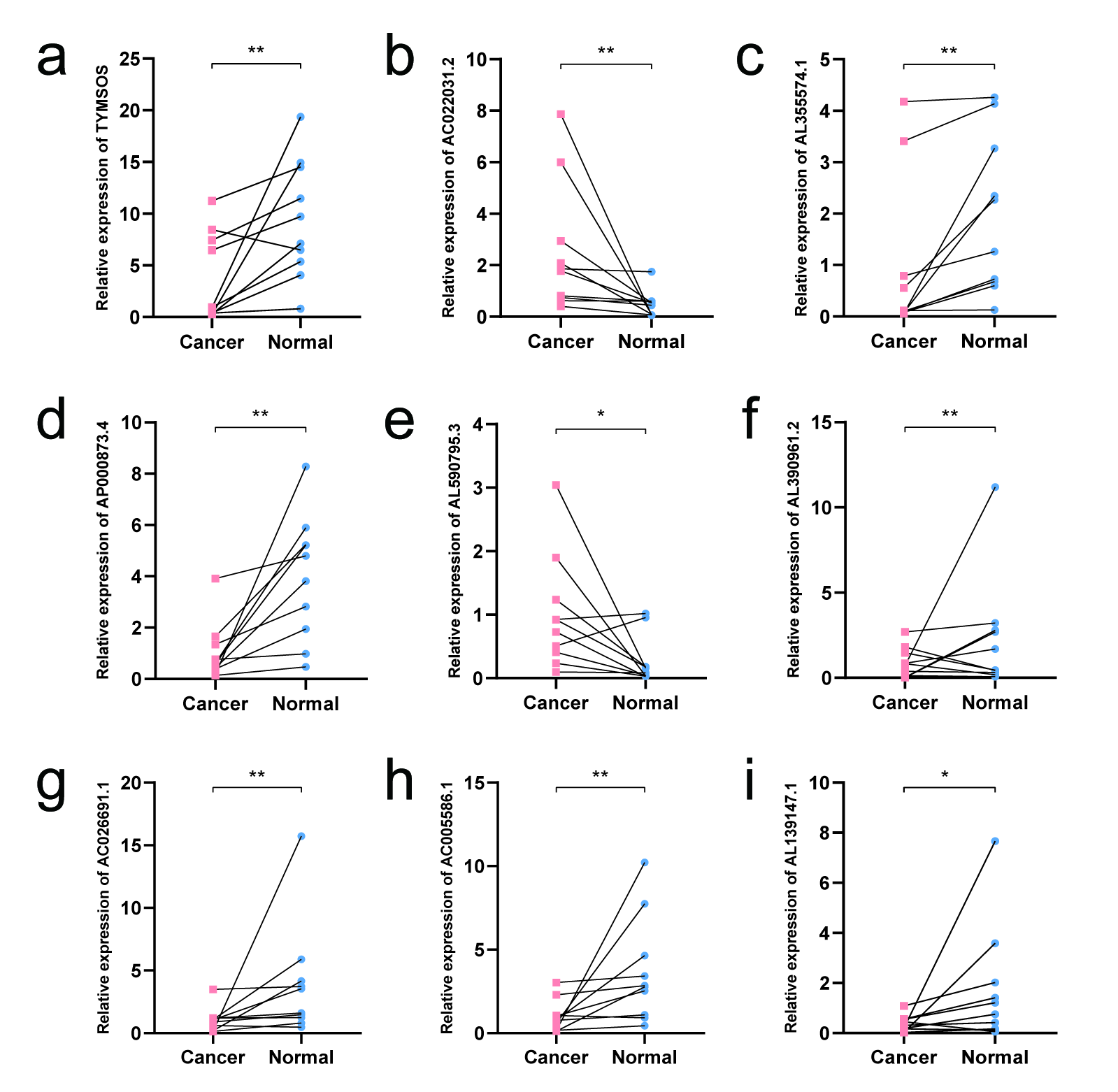

Supplement: Supplementary file 10 — Additional file 10: Figure S5. Expression of m6A-related lncRNAs in GC patients. Relative of RNA expression of lncRNAs between cancerous and adjacent normal tissues: (a) TYMSOS, (b) AC022031.2, (c) AL355574.1, (d) AP000873.4, (e) AL590705.3, (f) AL390961.2, (g) AC026691.1, (h) AC005586.1, and (i) AL139147.1. P < 0.05 * and P < 0.01 **. [file 12935_2021_2146_MOESM10_ESM.tif]

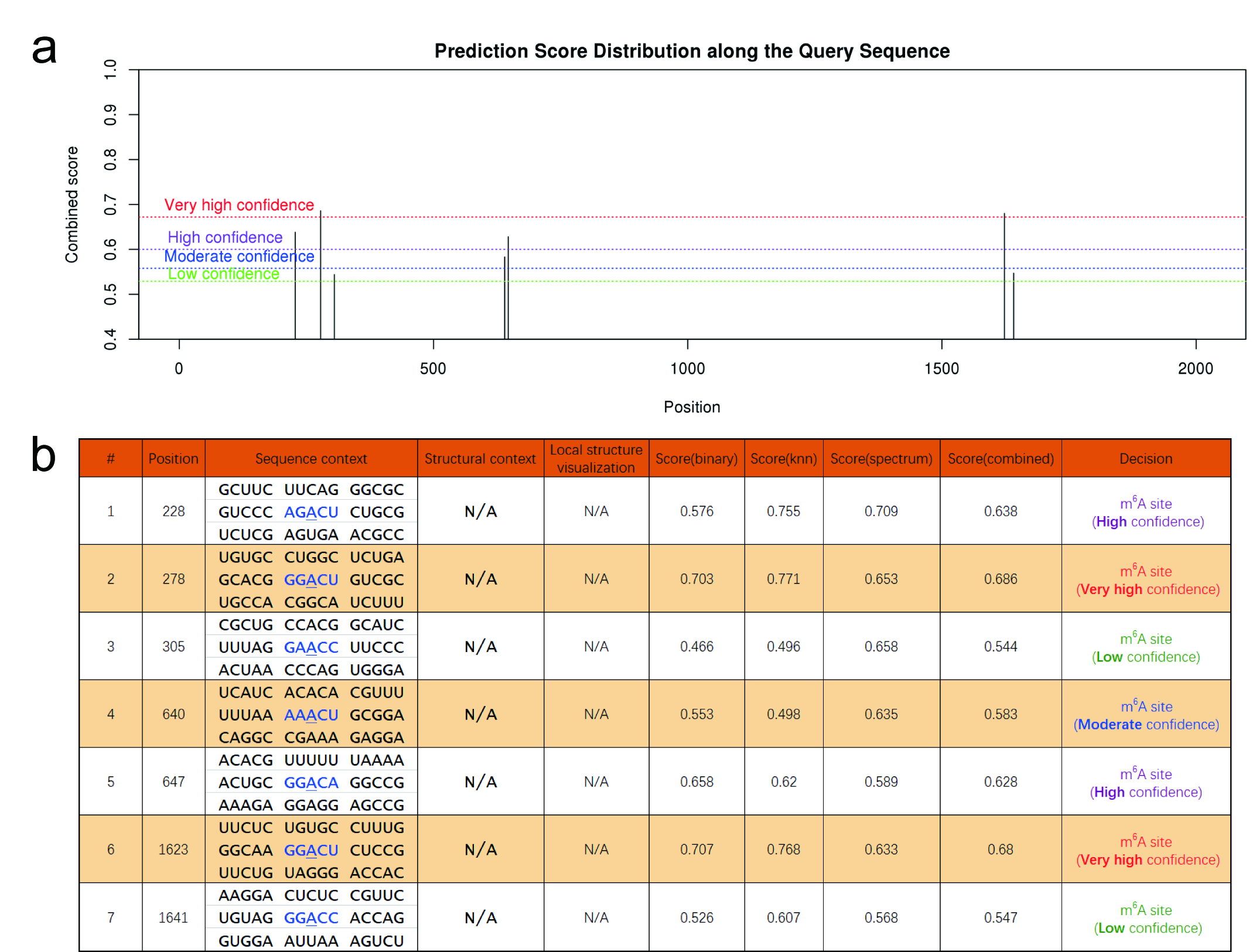

Supplement: Supplementary file 12 — Additional file 12: Figure S6. Potential m6A modification positions of lncRNA AC026691.1. (a) The underlying modification sites distributed along the sequence of lncRNA AC026691.1. (b) The detailed information about prediction positions. m6A, N6-methyladenosin; lncRNA, long noncoding RNA. [file 12935_2021_2146_MOESM12_ESM.tif]
