# Supplementary material for: Identification of a robust signature for clinical outcomes and immunotherapy response in gastric cancer: based on N6-methyladenosine related long noncoding RNAs
Source: Cancer Cell Int. 2021 Aug 16;21:432. doi: 10.1186/s12935-021-02146-w (PMC8365962; doi:10.1186/s12935-021-02146-w)
Supplement: Supplementary file 6 — Additional file 6: Table S4. Coefficient of LASSO model in this study. [file 12935_2021_2146_MOESM6_ESM.docx]

**Table S4. Coefficient of LASSO model in this study.**

| **LincRNA** | **Coefficients** |
| --- | --- |
| AP000873.4 | -0.363505962365076 |
| AC026691.1 | 0.478450097207464 |
| AC005586.1 | -0.272397879376476 |
| AL390961.2 | -0.22886401876192 |
| AL590705.3 | 0.387415856495822 |
| TYMSOS | -0.0585586582207713 |
| AL139147.1 | 0.47058085505868 |
| AC022031.2 | 0.180678428922293 |
| AL355574.1 | -0.108539848420546 |
