# Supplementary material for: Identification of a robust signature for clinical outcomes and immunotherapy response in gastric cancer: based on N6-methyladenosine related long noncoding RNAs
Source: Cancer Cell Int. 2021 Aug 16;21:432. doi: 10.1186/s12935-021-02146-w (PMC8365962; doi:10.1186/s12935-021-02146-w)
Supplement: Supplementary file 7 — Additional file 7: Table S5. Baseline of training and testing sets. [file 12935_2021_2146_MOESM7_ESM.docx]

**Table S5. Baseline of training and testing sets.**

|  | **test** | **train** | **p.overall** |
| --- | --- | --- | --- |
|  | ***N=184*** | ***N=187*** |  |
| Age | 66.1 (10.4) | 65.5 (10.8) | 0.563 |
| Gender: |  |  | 0.326 |
| FEMALE | 71 (38.6%) | 62 (33.2%) |  |
| MALE | 113 (61.4%) | 125 (66.8%) |  |
| futime | 1.48 (1.48) | 1.69 (1.50) | 0.188 |
| fustat: |  |  | 0.445 |
| 0 | 108 (58.7%) | 118 (63.1%) |  |
| 1 | 76 (41.3%) | 69 (36.9%) |  |
| Grade: |  |  | 0.103 |
| G1 | 7 (3.80%) | 3 (1.60%) |  |
| G2 | 56 (30.4%) | 78 (41.7%) |  |
| G3 | 116 (63.0%) | 102 (54.5%) |  |
| GX | 5 (2.72%) | 4 (2.14%) |  |
| Stage: |  |  | 0.534 |
| Stage I | 22 (12.0%) | 28 (15.0%) |  |
| Stage II | 52 (28.3%) | 59 (31.6%) |  |
| Stage III | 82 (44.6%) | 67 (35.8%) |  |
| Stage IV | 18 (9.78%) | 20 (10.7%) |  |
| unknow | 10 (5.43%) | 13 (6.95%) |  |
| T: |  |  | 0.494 |
| T1 | 9 (4.89%) | 9 (4.81%) |  |
| T2 | 37 (20.1%) | 41 (21.9%) |  |
| T3 | 78 (42.4%) | 89 (47.6%) |  |
| T4 | 57 (31.0%) | 43 (23.0%) |  |
| unknow | 3 (1.63%) | 5 (2.67%) |  |
| M: |  |  | 0.632 |
| M0 | 165 (89.7%) | 163 (87.2%) |  |
| M1 | 12 (6.52%) | 13 (6.95%) |  |
| MX | 7 (3.80%) | 11 (5.88%) |  |
| N: |  |  | 0.528 |
| N0 | 48 (26.1%) | 60 (32.1%) |  |
| N1 | 49 (26.6%) | 48 (25.7%) |  |
| N2 | 40 (21.7%) | 34 (18.2%) |  |
| N3 | 40 (21.7%) | 34 (18.2%) |  |
| NX | 7 (3.80%) | 11 (5.88%) |  |
