# Supplementary material for: Identification of a robust signature for clinical outcomes and immunotherapy response in gastric cancer: based on N6-methyladenosine related long noncoding RNAs
Source: Cancer Cell Int. 2021 Aug 16;21:432. doi: 10.1186/s12935-021-02146-w (PMC8365962; doi:10.1186/s12935-021-02146-w)
Supplement: Supplementary file 11 — Additional file 11: Table S6. Correlation of LncRNA AC026691.1 and m6A-related genes. [file 12935_2021_2146_MOESM11_ESM.docx]

**Table S6. Correlation of LncRNA AC026691.1 and m6A-related genes**

| **m6A related genes** | **Correlation coefficient** | **P value** | **Regulation** |
| --- | --- | --- | --- |
| **FTO** | **0.507956747** | **5.43E-26** | **positive** |
| METTL16 | 0.307915713 | 1.12E-09 | positive |
| ALKBH5 | 0.168777363 | 0.001034338 | positive |
| METTL14 | 0.15551299 | 0.002529123 | positive |
| IGFBP3 | 0.116101964 | 0.024550208 | positive |
| WTAP | -0.171197403 | 0.000872325 | negative |
| LRPPRC | -0.172111696 | 0.000817472 | negative |
| YTHDF1 | -0.172117758 | 0.000817119 | negative |
| RBMX | -0.240593607 | 2.44E-06 | negative |
| YTHDF2 | -0.24419659 | 1.70E-06 | negative |
| HNRNPA2B1 | -0.244821764 | 1.60E-06 | negative |
| HNRNPC | -0.304795095 | 1.67E-09 | negative |
